# DEPTOR in Skeletal Development and Diseases

**DOI:** 10.3389/fgene.2021.667283

**Published:** 2021-05-27

**Authors:** Jose Miguel Perez-Tejeiro, Fabiana Csukasi

**Affiliations:** ^1^Department of Cell Biology, Genetics and Physiology, Faculty of Sciences, IBIMA, University of Málaga, Málaga, Spain; ^2^Biomaterials and Nanomedicine (CIBER-BBN), Andalusian Centre for Nanomedicine and Biotechnology-BIONAND, Networking Biomedical Research Center in Bioengineering, Málaga, Spain

**Keywords:** DEPTOR, mTOR, skeleton, bone, cartilage

## Abstract

Discovered in 2009, the DEP-domain containing mTOR-interacting protein, DEPTOR, is a known regulator of the mechanistic target of rapamycin (mTOR), an evolutionarily conserved kinase that regulates diverse cellular processes in response to environmental stimuli. DEPTOR was originally identified as a negative regulator of mTOR complexes 1 (mTORC1) and 2 (mTORC2). However, recent discoveries have started to unravel the roles of DEPTOR in mTOR-independent responses. In the past few years, mTOR emerged as an important regulator of skeletal development, growth, and homeostasis; the dysregulation of its activity contributes to the development of several skeletal diseases, both chronic and genetic. Even more recently, several groups have reported on the relevance of DEPTOR in skeletal biology through its action on mTOR-dependent and mTOR-independent pathways. In this review, we summarize the current understanding of DEPTOR in skeletal development and disease.

## A Brief Overview of DEPTOR

The DEP-domain containing mTOR-interacting protein (DEPTOR), also known as DEP-domain containing 6 (DEPDC6), has gained more attention from the scientific community since its discovery in 2009 due to the increasing amount of data revealing its crucial role in diverse biological processes and pathological conditions. Early studies discovered its function as a member of the mechanistic target of rapamycin (mTOR) complexes, mTORC1, and mTORC2 (Peterson et al., 2009). However, recent studies have reported some evidence that points to possible mTOR-independent functions of DEPTOR (Catena et al., 2016; Li et al., 2020).

**FIGURE 1:**
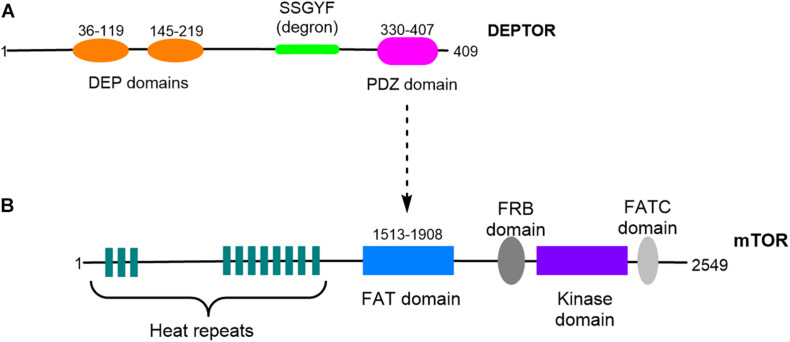
Schematic representation of DEPTOR and mTOR structures. **(A)** DEPTOR protein presents two DEP domains organized in tandem and a PDZ domain. A conserved linker sequence, DEPTOR degron, is located between the DEP and the PDZ domains. **(B)** DEPTOR PDZ domain is responsible for the interaction with mTOR FAT domain.

DEPTOR is encoded by a gene located on chromosome 8 (cytogenetic location 8q24.12) and is only present in vertebrates, sharing a high degree of homology between different organisms (Peterson et al., 2009). DEPTOR is a DEP-domain protein (Consonni et al., 2014), a diverse family of proteins with different mechanisms of action that are key in the control of signal transduction pathways (Consonni et al., 2014). DEPTOR’s structure is characterized by the presence of two different highly conserved regions: two DEP domains in tandem at the N-terminal region (residues 36–119 and 145–219) and a PDZ domain at the C-terminal region (residues 330-407). Connecting the second DEP domain and the PDZ domain is a consensus sequence, SSGYFS (Peterson et al., 2009; Figure 1). The DEP domains were first identified in three proteins: disheveled, pleckstrin, and egg-laying defective protein 10 (Consonni et al., 2014). A well-described function of the DEP domains is plasma membrane anchoring; however, the function of the DEPTOR DEP domains remains unclear. The PDZ domain is found in a number of proteins and is involved in protein–protein interaction; the DEPTOR PDZ domain is responsible for DEPTOR interaction with the FAT domain of mTOR (Peterson et al., 2009; Grabiner et al., 2014; Ghosh et al., 2015; Figure 1). The conserved linker region located between the second DEP domain and the PDZ domain is known as DEPTOR degron due to the presence of multiple phosphorylation sites involved in DEPTOR degradation (Duan et al., 2011; Gao et al., 2011; Zhao et al., 2011). Indeed the posttranslational control of DEPTOR levels seems to be the preferred mechanism of regulation of DEPTOR. DEPTOR protein is degraded by the proteasome after serum-induced phosphorylation of 13 serine and threonine residues (Peterson et al., 2009). Mechanistically, DEPTOR degradation is controlled by the Skp1-Cul1-Fbox protein (SCF) complex, which binds to βTrCP, an E3 ubiquitin ligase involved in other protein degradation processes (Duan et al., 2011; Gao et al., 2011; Zhao et al., 2011).

*DEPTOR* is also regulated at the transcriptional level, but contrary to its posttranslational regulation, the transcriptional control of *DEPTOR* is more tissue and environment specific. Several growth factors such as transforming growth factor β (TGFβ) and epidermal growth factor (EGF) have been associated with changes in *DEPTOR*, decreasing its expression (Das et al., 2014; Zhou et al., 2016). Hormones also play a role in the regulation of *DEPTOR* gene expression; androgen and estrogen receptors have been suggested as negative and positive regulators of *DEPTOR*, respectively (Kanno et al., 2015; Parvani et al., 2015). Glucocorticoids such as dexamethasone and corticosterone have also been associated with the transcriptional control of *DEPTOR*, positively regulating its expression (Laplante et al., 2012). Cellular stress is another condition known to regulate *DEPTOR* mRNA levels. Rb-binding protein Che-1 (Che-1), a transcriptional regulator that responds to DNA damage, hypoxia, and glucose deprivation, promotes the expression of *DEPTOR* in cancer cells under hypoxia (Desantis et al., 2015). Finally, two transcriptional regulators, Six4 and Baf60c, coordinately stimulate *Deptor* expression in muscle cells (Meng et al., 2013).

As previously mentioned, DEPTOR was first identified as an mTOR-interacting protein (Peterson et al., 2009). mTOR is an evolutionarily conserved serine–threonine kinase whose role is to integrate different stimuli from the environment and translate them into a variety of cellular responses (Sarbassov et al., 2005). mTOR constitutes the catalytic subunit of two different complexes, mTOR complex 1 (mTORC1) and mTOR complex 2 (mTORC2). The two complexes have distinct functions and respond to different environmental stimuli. mTORC1 is composed of the proteins regulatory-associated protein of mTOR (RAPTOR), proline-rich AKT substrate 40 kDa (PRAS40), mammalian lethal with Sec13 protein 8 (mLST8, also known as GβL), and DEPTOR, is activated in response to nutrients, amino acids, growth factors, and energy sufficiency, and plays a pivotal role in the regulation of cell growth and proliferation by promoting lipid and protein synthesis through phosphorylating eukaryotic translation initiation factor 4E-binding protein 1 (4EBP1) and ribosomal protein S6Kinase beta-1 (S6K1). mTORC1 also inhibits autophagy by phosphorylating and suppressing Unc-51 like autophagy activating kinase 1 (ULK1) activity. On the contrary, the major role of mTORC2 is to control cell survival in response to growth factors through the activation of protein kinase B (AKT). mLST8/GβL and DEPTOR are also found in mTORC2, whereas rapamycin-insensitive companion of mTOR (RICTOR), mammalian stress-activated protein kinase interacting protein (mSIN1), and protein observed with Rictor-1 (PROTOR) are its unique components (Guertin and Sabatini, 2007).

Being a member of the two mTOR complexes, DEPTOR plays a role in the regulation of both of them. DEPTOR was originally described as a negative regulator of mTORC1 and mTORC2 because of the results obtained in loss-of-function and kinase assay experiments, which showed decreased phosphorylation (and therefore activity) of S6K1 and AKT (Peterson et al., 2009), outputs of mTORC1 and mTORC2, respectively. However, when DEPTOR was overexpressed in cells, it resulted in the inhibition of mTORC1 activity and also in the activation of mTORC2. Therefore, another model was explained, in which the inhibition of mTORC1 by DEPTOR resulted in an indirect effect in mTORC2 through the release of its inhibition on PI3K, which controls mTORC2 (Peterson et al., 2009). This “feedback model” seems to be supported by most of the work that have been published (Caron et al., 2018).

As expected, DEPTOR has been implicated in several of the pathways regulated by mTORC1 and mTORC2 such as cell proliferation, autophagy, and apoptosis, but evidence is emerging on the roles of DEPTOR that are independent of mTOR. In this review, we will specifically focus on the role of DEPTOR in skeletal development and disease, which started to emerge in 2016 when *Deptor* was associated with bone mineral density (BMD) (Reyes Fernandez et al., 2016). Given that DEPTOR is an important member of the mTOR complexes, we will briefly summarize some of the major discoveries that have revealed mTOR as an important regulator of skeletal development, growth, and homeostasis. However, a more detailed view of the mTOR pathway in cartilage and bone has been recently reviewed (Chen and Long, 2018; Shen et al., 2018). A very detailed review of the roles played by DEPTOR in other tissues and diseases was recently published by Caron et al. (2018).

## mTOR Pathway in Skeletal Development and Homeostasis

Skeletal development occurs through two distinct processes, endochondral and membranous ossification. In membranous ossification, mesenchymal progenitors condense and progress almost directly to the bone, whereas endochondral ossification is characterized by the formation of a cartilaginous intermediate that will ultimately be replaced by bone. The chondrocytes that form the cartilage initially proliferate and then undergo maturation, leading to the sequential formation of prehypertrophic, early hypertrophic, and late hypertrophic chondrocytes. Finally, blood vessels invade the cartilage, bringing osteoblasts and osteoclasts which will be responsible for bone formation and resorption, respectively. The bones of the skull, lateral clavicle, and pubis form *via* membranous ossification, whereas endochondral ossification forms the appendicular skeleton and some parts of the axial skeleton. During the process of endochondral bone formation, chondrocytes acquire diverse shapes and express different genes that characterize the different stages of chondrocyte development. Several signaling pathways act in concert to regulate skeletal development such as wingless-related integration site (WNT), TGFβ, bone morphogenetic protein (BMP), parathyroid hormone (PTH), and parathyroid hormone-related peptide (PTHrP) (Hall and Miyake, 2000; Kronenberg, 2003; Kozhemyakina et al., 2015).

A considerable number of publications have addressed the role played by mTOR signaling in skeletal development. The first discoveries linking mTOR with cartilage formation were made through the study of the application of the mTORC1 chemical inhibitor rapamycin, which inhibited the formation of cartilage nodules from limb cells (Oh et al., 2001; Phornphutkul et al., 2008; Jiang et al., 2017). More recently, mouse genetic studies have implicated RAPTOR in chondrogenesis. Deletion of *Raptor* at the early limb bud stage using *Prx-Cre* impaired skeletal growth through decreased chondrocyte size and matrix production and delay in chondrocyte hypertrophy and bone formation (Chen and Long, 2014). Deletion of the mTORC1 negative regulator tuberous sclerosis complex (*Tsc1*) induced increased chondrocyte proliferation and decreased differentiation, resulting in a chondrodysplasia phenotype (Yan et al., 2016). On the contrary, decreased mTORC2 signaling, through the deletion of *Rictor* using *Prx-Cre*, had a less pronounced effect in the skeleton, which was a consequence of delayed chondrocyte hypertrophy only (Chen et al., 2015).

More studies have implicated mTOR in bone formation and homeostasis. The genetic ablation of *Raptor* in mouse preosteoblasts using a conditional *Osx-Cre* system resulted in decreased bone formation due to deficient matrix synthesis and mineralization (Huang et al., 2015). A transcriptomic analysis of the mutant mice demonstrated that the osteoblasts showed a phenotype of arrested differentiation, supporting that mTORC1 promotes the transition from preosteoblasts to mature osteoblasts (Huang et al., 2015). Interestingly, deletion of *Tsc1* in neural crest-derived cells resulted in sclerotic craniofacial bone lesions (Fang et al., 2015), similar to the ones observed in patients with tuberous sclerotic syndrome, a disease mainly characterized by the presence of benign tumors in the skin, brain, kidney, and heart but in which around half of the patients develop sclerotic bone lesions. The deletion of *Tsc1* resulted in an increased osteoblast population, leading to increased bone mass. In other studies, deletion of *Tsc1* and *Tsc2* in osteoblasts resulted in higher osteoblast proliferation but impaired osteoblast differentiation (Fang et al., 2015).

With respect to osteoclastogenesis, the role of mTOR is less well understood, and some reports have been contradictory. For example, one group reported that the conditional deletion of *Raptor* and of *Tsc1* in osteoclast precursors using *Lyz-Cre* resulted in enhanced and impaired osteoclastogenesis, respectively (Zhang Y. et al., 2017). However, another group reported high bone mass due to reduced osteoclastogenesis after the conditional deletion of *Raptor* in osteoclasts with *Ctsk-Cre* (Dai et al., 2017). The different results obtained by the two groups are probably due to the fact that *Raptor* was deleted at different stages of the osteoclasts, indicating that the role of mTORC1 might be stage specific, similar to what was observed in the mTORC1 role in cartilage formation (Yan et al., 2016).

## DEPTOR in Cartilage and Bone Development and Pathologies

Recent data from different groups has brought attention to the role played by DEPTOR in skeletal development and diseases, a role previously unexplored in the field of skeletal biology. Specifically, DEPTOR has been implicated in cartilage and bone biology, and its dysregulation results in the development of several skeletal diseases, both chronic and genetic.

**FIGURE 2:**
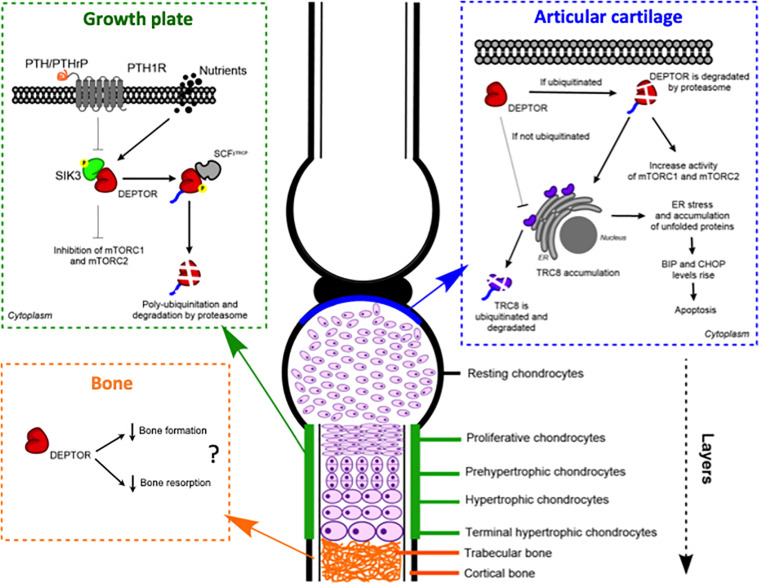
DEPTOR plays roles in different skeletal tissues: growth plate, articular cartilage, and bone. Models of the proposed molecular mechanisms of action of DEPTOR on the different tissues.

In 2018, we reported for the first time aberrant DEPTOR levels in two skeletal genetic disorders, spondyloepimethapyseal dysplasia, Krakow type and Jansen metaphyseal chondrodysplasia (JMC) (Csukasi et al., 2018). These two disorders share some common features and are caused by mutations in salt-inducible kinase 3 (*SIK3*) (later named as spondyloepimethapyseal dysplasia, Krakow type) and in the PTH and PTHrP receptor, parathyroid hormone 1 receptor (*PTH1R*) genes, respectively (JMC). We observed a decreased activity of mTORC1 and mTORC2 due to the accumulation of DEPTOR, which was also found in JMC. Our data demonstrated that SIK3 is an essential positive regulator of mTOR signaling that functions by triggering DEPTOR degradation by the proteasome in response to PTH/PTHrP signaling during skeletogenesis (Csukasi et al., 2018; Figure 2).

We showed that the accumulation of DEPTOR resulted in a disorganization of the growth plate in JMC patients, which was characterized by a severe reduction in the number of hypertrophic chondrocytes. Also noticeable was the ectopic invasion of bone into the cartilaginous growth plate. Analysis of the localization of DEPTOR protein in healthy human growth plates determined that DEPTOR was mostly expressed in resting and proliferative zones and decreased in hypertrophic chondrocytes. In contrast, a decreased expression of DEPTOR was not observed in the hypertrophic zone of JMC patients’ growth plates, supporting that increased DEPTOR levels compromise the commitment of chondrocytes to hypertrophy (Csukasi et al., 2018; Figure 2).

In support of a crosstalk between mTORC1 and PTHrP signaling in the skeleton, by analyzing a chondrocyte-specific TSC1 KO mouse, another group demonstrated that mTORC1 regulates *PTHrP* transcription and that this control is essential to coordinate chondrocyte proliferation and differentiation (Yan et al., 2016). Mechanistically, mTORC1 control over *PTHrP* gene expression levels is achieved through phosphorylation-dependent nuclear translocation of GLI2. Later work from the same group reported that cartilage-specific TSC1 KO mice showed decreased levels not only of PTHrP but also of its receptor, PTH1R (Zhang H. et al., 2017). They also showed aberrant extracellular signal-regulated kinase (ERK) signaling in KO mice. Moreover, the chemical inhibition of ERK restored the PTH1R levels; therefore, they concluded that mTORC1 inhibits PTH1R expression in chondrocytes through the activation of ERK signaling (Zhang H. et al., 2017). However, no clear mechanism of action was indicated. Unfortunately, these two works did not address the specific role played by DEPTOR in the process.

Another work linking DEPTOR with cartilage development in the growth plate was recently reported, demonstrating a connection between DEPTOR and articular cartilage (Li et al., 2020). They determined that the DEPTOR levels were inversely correlated with the increase in cartilage damage in articular chondrocytes in an osteoarthritis (OA) mouse model. Furthermore, they found decreased levels of DEPTOR in OA patient cartilage. Finally, they detected a gradual reduction in the levels of DEPTOR in mice aged 4, 12, and 24 months. To confirm the role of DEPTOR in OA development, they specifically deleted *Deptor* in cartilage using *Col2a1-Cre*. The DEPTOR-KO mice did not exhibit any obvious skeletal abnormalities, indicating that the specific deletion of *Deptor* in chondrocytes does not produce major defects in skeletal development. However, traumatic OA surgery in DEPTOR-KO mice resulted in increased damage in the articular cartilage of mutant mice. The defects observed were restored upon overexpression of *Deptor* (Li et al., 2020).

The authors concluded that the DEPTOR effects on OA progression were mTOR independent because the treatment of DEPTOR-KO mice with two different mTOR inhibitors, rapamycin and AZD8055, had no effect on the mice phenotype (Li et al., 2020). However, they reported a decrease in mTORC1 and mTORC2 activity, so the conclusion that the effect of DEPTOR is mTOR independent should be taken with caution. Furthermore, partial inhibition of mTOR activity with the use of chemicals leads to an unbalanced mTORC1/2 signaling, characterized by inhibition of mTORC1 and activation of mTORC2 through the release of the negative feedback of mTORC1 on PI3K (Peterson et al., 2009). Therefore, the effects that mTOR chemical inhibitors can have on DEPTOR are unclear. Supporting mTOR involvement in OA, mTORC1 activation by genetic ablation of *Tsc1* using *Col2a1-Cre* resulted in the accelerated progression of OA and also a more severe OA phenotype (Zhang H. et al., 2017). The knockout of mTOR in mice using *Col2a1-Cre* also protected from OA progression (Zhang et al., 2015). Hyperactivation of mTOR was found in human OA cartilage and mice with injury-induced OA (Zhang et al., 2015; Zhang H. et al., 2017). On the contrary, chemical or genetic inhibition of mTORC1 attenuated OA in animal models (Caramés et al., 2012; Matsuzaki et al., 2014; Cheng et al., 2016). The mechanism by which mTOR controls OA remains obscure, but some evidence suggest that it might be related to the regulation of autophagy (Caramés et al., 2010; Vasheghani et al., 2015; Zhang et al., 2015; Cheng et al., 2016).

A different mechanism was suggested for DEPTOR control of OA progression. Using a proteomic approach. Li et al. (2020) found that the levels of translocation in renal cancer from chromosome 8 (TRC8), a ubiquitin ligase located in the ER membrane and involved in ER-associated protein degradation (Araki and Nagata, 2011; Christianson and Ye, 2014), were increased in DEPTOR-KO mice (Li et al., 2020). DEPTOR interacted with TRC8 in ATDC5 cells, and the interaction was stronger in the articular chondrocytes of the OA mouse model. Moreover, TRC8 ubiquitination was significantly decreased in DEPTOR knockdown (KD) cells, suggesting that the DEPTOR mechanism of action is to promote TRC8 auto-ubiquitination in articular cartilage during OA (Li et al., 2020). Because recent evidence showed an increased expression of ER stress markers in TRC8 KO mice (Chang et al., 2015), the authors evaluated whether ER stress was also affected in DEPTOR KD cells. They found increased levels of binding immunoglobulin protein (BiP) and CAAT-enhancer-binding protein homologous protein (CHOP) in the cells, but no data was presented using DEPTOR KO mice (Figure 2). Furthermore, treatment of DEPTOR KO mice with the ER stress inhibitor, 4-phenyl butyric acid (4-PBA), attenuated cartilage destruction in both WT and DEPTOR KO mice (Li et al., 2020). Interestingly, treatment of TSC1 KO mice with 4-PBA did not restore the OA phenotype of mutant mice (Zhang H. et al., 2017), indicating that the mechanisms by which DEPTOR and mTORC1 control the progression of the disease are different and that the DEPTOR role might be mTOR independent as suggested by Li et al. (2020). In support of a role of DEPTOR in ER stress, another study in multiple myeloma (MM), a cancer in which DEPTOR has a crucial role, also found increased levels of BiP and CHOP in DEPTOR KD MM cells. Besides this, they found increased levels of DEPTOR after treatment with the ER stress inducers, tunicamycin, and brefeldin A. However, the mechanism described in MM involved DEPTOR acting in the nucleus to control the transcription of BiP and CHOP (Catena et al., 2016).

Several evidence suggest an implication of DEPTOR in bone formation and resorption (Figure 2). To our knowledge, the first study to ever link DEPTOR with the skeleton and, in particular, with bone was reported in 2016 (Reyes Fernandez et al., 2016). The authors performed a quantitative trait loci (QTL) analysis to identify the genetic loci that regulate calcium (Ca) and bone metabolism. For the study, they used 51 mouse BXD (established from an intercross between C57BL/6J, B6 and DBA/2J, DBA; Peirce et al., 2004) recombinant inbred lines and tested two diets with different concentrations of Ca: 0.5%, which is considered adequate, and 0.25%, as a low-Ca diet. The *Deptor* mRNA level was positively correlated to changes in BMD in mice on the low-Ca diet, providing a novel link between Ca metabolism and DEPTOR (Reyes Fernandez et al., 2016). Interestingly, a connection between vitamin D, which is involved in maintaining Ca homeostasis in bone, and DEPTOR levels has been observed. A vitamin D receptor KO mouse showed low levels of *Deptor* mRNA, although no proof of a direct effect of the receptor on DEPTOR was provided (Lisse et al., 2010). Ca signaling has also been implicated in mTOR activity. In response to amino acids, S6K1 is inhibited by a Ca chelator, whereas the release of intracellular Ca activates mTORC1. Moreover, amino acid-induced mTORC1 activation is inhibited by calmodulin (CaM) antagonists and through a CaM siRNA (Gulati et al., 2008). Proper lysosomal Ca release is also required for mTORC1 activation and is achieved by a direct interaction between CaM and mTOR (Li et al., 2016). A biological consequence of the dysregulation of Ca and mTOR/DEPTOR has not been established; future work will hopefully fill this gap of knowledge. Another interesting perspective is a possible crosstalk between Ca and PTH in DEPTOR regulation, given that PTH is secreted in response to low levels of Ca.

A recent genome-wide association study (GWAS) performed to identify new genomic loci associated with BMD identified *DEPTOR* at the genome-wide significance level. The analysis was performed in over 490,000 participants from the United Kingdom biobank cohort which analyzed estimated heel BMD (eBMD) and from 30 studies analyzing total body BMD (TB-BMD). Integration of this information identified 13 novel genomic loci, nine for eBMD and four for TB-BMD; *DEPTOR* was located in the eBMD group (Liu et al., 2020).

Pereira et al. (2020) recently reported on a role of DEPTOR in bone resorption. Taking advantage of MMnet, a trans-regulated gene network of macrophage multinucleation, which is enriched for GWAS variants associated with bone phenotypes, they identified several genes that regulate bone function. One of the genes was *Deptor*. Analysis of DEPTOR KO mice revealed low bone mass and vertebral stiffness. Knockdown of DEPTOR in human osteoclasts stimulated multinucleation and resorption, suggesting that the decrease in bone mass caused by *Deptor* depletion might be due to DEPTOR action on bone resorption and not on bone formation. Interestingly, knockdown or deletion of phosphoinositide 3-kinase (PI3K) (a positive regulator of mTORC1) resulted in the opposite phenotype, decreased multinucleation and resorption, and increased bone mass, consistent with the opposite effects of DEPTOR and PI3K on mTORC1 (Pereira et al., 2020). Unfortunately, because the purpose of this work was to functionally validate MMet in osteoclast multinucleation and bone mass and it therefore involved the evaluation of 12 MMet genes using mouse genetics, the effects of global DEPTOR KO in the skeleton were not evaluated in much detail. Whole-body DEPTOR KO and overexpressor mice have also been described by a previous work. Although no obvious skeletal defects were reported, a thorough study of the skeleton was not performed, and therefore we cannot rule out skeletal defects in these models (Laplante et al., 2012).

Finally, an analysis of an ovariectomized mouse, usually used as a model of osteoporosis, revealed elevated levels of DEPTOR compared to control, suggesting a positive correlation between DEPTOR and osteoporosis (Chen S. et al., 2018). Interestingly, the genetic ablation of *Tsc1* in osteoclasts with the use of *Lyz2-Cre* prevented bone loss in a mouse model of osteoporosis (induced by the receptor activator of nuclear factor kappa-B ligand injections). On the contrary, no effect on bone resorption was found in TSC1 CKO under normal conditions, suggesting that mTORC1 plays a role in bone resorption under pathological conditions (Hiraiwa et al., 2019).

## DEPTOR in Skeletal Progenitors Differentiation

Another possible role of DEPTOR in skeletal formation involves the regulation of stem cell differentiation. MSC is a population of multipotent stem cells located in the bone marrow and other adult tissues, which are able to differentiate into different skeletal tissues such as bone, cartilage, and fat in response to different signals from the environment (Cook and Genever, 2013). Recently, Chen S. et al. (2018) reported a decreased expression of DEPTOR during osteogenic differentiation of primary human bone marrow MSC (hBMSC), which was consistent with an upregulation of bone markers such as runt-related transcription factor 2, alkaline phosphatase (*ALP*), and osteocalcin. Furthermore, the knockdown of DEPTOR resulted in increased ALP activity and mineralization, indicating that DEPTOR is a negative regulator of osteogenesis (Chen S. et al., 2018). Although this might sound contradictory with the fact that DEPTOR KO mice show decreased bone mass. Pereira et al. (2020) showed that the knockdown of DEPTOR in osteoclasts resulted in increased bone resorption. From these two studies, we can speculate that the role of DEPTOR might be to inhibit the osteogenesis of skeletal precursors and also to inhibit bone resorption in adult cells, which could explain the decreased bone mass in DEPTOR KO mice and the increased osteogenic capacity of DEPTOR KD MSC. In support of a negative regulation of DEPTOR in osteogenesis, inactivation of mTORC1 by treatment of mouse BMSC with rapamycin decreased their osteogenic differentiation (Singha et al., 2008; Xian et al., 2012), whereas increased mTORC1 activity by insulin-like growth factor 1 treatment, stimulated osteogenesis (Xian et al., 2012). Besides this, the deletion of *Rictor* in BMSC also decreased osteogenic differentiation (Sen et al., 2013; Chen et al., 2015; Martin et al., 2015). Regarding the mechanism by which DEPTOR inhibits osteogenesis, an increased expression of the long non-coding RNA maternally expressed gene 3 (*MEG3*) was found in DEPTOR KD hBMSC cells (Chen S. et al., 2018). Some evidence show that MEG3 promotes osteogenesis while inhibiting adipogenesis (Zhuang et al., 2015; Li, 2017). The knockdown of MEG3 in DEPTOR-depleted cells restored ALP activity and mineralization to normal levels and also reduced the *BMP4* expression levels. By performing ChIP-qPCR, the authors concluded that DEPTOR directly binds to the promoter of *BMP4* to regulate its transcription, a finding that suggests a role of DEPTOR as a transcriptional regulator in the nucleus (Chen S. et al., 2018). In line with this evidence of DEPTOR acting as a transcriptional regulator, Catena et al. (2016) suggested that DEPTOR exerts a direct control on the transcription of ER stress genes in MM cells by binding their promoter regions. To our knowledge, these two works are the only ones so far that have reported a role of DEPTOR in the nucleus, and they constitute a promising new view of the DEPTOR mode of action, but it requires further investigation to fully address whether the effects of DEPTOR in transcription are direct or indirect.

Although DEPTOR control over the adipogenic lineage commitment of BMSC has not been assessed, DEPTOR involvement in adipogenesis is well documented. Overexpression of DEPTOR in mice promotes white adipose tissue accumulation when the animals are fed a high-fat diet. Transgenic mice showed an increased expression of target genes of the adipogenesis regulator peroxisome proliferator-activated receptor gamma (PPARY) (Laplante et al., 2012). Furthermore, a section on chromosome 15 that includes *Deptor* was identified as part of the *Fob3a* QTL linked to obesity in mice (Stylianou et al., 2005). The DEPTOR levels also increased during adipogenic differentiation of mouse embryonic fibroblasts (MEFs) and 3T3-L1 cells, indicating that DEPTOR promotes adipogenesis. Furthermore, the increase in DEPTOR expression correlated to the activation of AKT and PPARY, which resulted in diminished mTORC1-mediated inhibition of insulin signaling (Laplante et al., 2012). This work revealed for the first time that DEPTOR is a positive regulator of adipogenesis. It would be interesting to determine if this regulation is also true in MSC; considering that DEPTOR acts by inhibiting the differentiation of MSC toward osteogenesis (Chen S. et al., 2018), it would be reasonable to assume that its promotion of adipogenesis will also be found in MSC cells. The fact that the deletion of *Rictor* in BMSC resulted in increased adipogenesis (Sen et al., 2013; Chen et al., 2015; Martin et al., 2015) also supports the involvement of mTOR-DEPTOR in this process.

## Concluding Remarks

These studies altogether provide a hint that DEPTOR functions to regulate cartilage and bone formation and homeostasis, probably under specific environmental and pathological situations: ER stress, Ca deprivation, aberrant PTH signaling, osteoporosis, osteoarthritis, and genetic diseases. On the contrary, it seems clear that, under normal conditions, DEPTOR is probably not critical for proper skeletal development and maintenance. However, much work is required to fully understand how DEPTOR acts to control and coordinate all these processes and to identify new pathways where DEPTOR might function. Furthermore, it is becoming more and more clear that DEPTOR is a lot more than only an mTOR inhibitor; this is probably only one of its multiple functions which happened to be the first discovered. In conclusion, it is very exciting to be a part of a growing community that is dedicated to unraveling the multifaceted role of DEPTOR in a variety of biological processes and, maybe in the future, use it as a target for the development of new therapeutics of several diseases that show the dysregulation of DEPTOR.

## Author Contributions

JP-T wrote the introduction and made the figures. FC wrote the rest of the manuscript. Both authors approved the submitted version.

## Conflict of Interest

The authors declare that the research was conducted in the absence of any commercial or financial relationships that could be construed as a potential conflict of interest.
